# Youth susceptibility to tobacco use: is it general or specific?

**DOI:** 10.1186/s12889-021-11956-6

**Published:** 2021-10-21

**Authors:** Hui G. Cheng, Pavel N. Lizhnyak, Natasha A. Knight, Andrea R. Vansickel, Edward G. Largo

**Affiliations:** Altria Client Services LLC, 601 E. Jackson, Richmond, VA 23219 USA

**Keywords:** Tobacco prevention, Youth, Susceptibility to tobacco use, Prospective study

## Abstract

**Background:**

Susceptibility to tobacco use predicts tobacco use onset among youth. The current study aimed to estimate the extent of overlap in susceptibilities across various tobacco products, investigate sociopsychological correlates with susceptibilities, and examine whether the relationship linking susceptibility with the onset of use is product-specific or is accounted for by a general susceptibility-onset relationship.

**Methods:**

The study population consisted of US youth 12–17 years old who had never used a tobacco product, sampled in the longitudinal Population Assessment of Tobacco and Health study wave 4 (Dec. 2016-Jan. 2018; *n* = 10,977). Tobacco product-specific susceptibility at wave 4 was assessed via questions about curiosity, likelihood to try, and likelihood of use if a best friend offered. The onset of use of various tobacco products was defined as first use occurring between the wave 4 and wave 4.5 (Dec. 2017-Dec. 2018) assessments (*n* = 8841). Generalized linear regression and structural equation models were used for data analysis.

**Results:**

There is a large degree of overlap in susceptibilities across tobacco products (65% of tobacco-susceptible youth were susceptible to more than one tobacco product). Tobacco-susceptible youths were more likely to have recently used cannabis, consumed alcohol, or to have been associated with tobacco-using peers. Structural equation models suggest that the susceptibility-onset relationship largely operates in a non-product-specific manner after accounting for the general susceptibility-to-tobacco-onset relationship.

**Conclusions:**

Youth susceptibility to tobacco use overlaps widely across different tobacco products and other risky behaviors. Findings from this study support a holistic approach towards the prevention of risk behaviors, supplemented by product-specific strategies when needed.

**Supplementary Information:**

The online version contains supplementary material available at 10.1186/s12889-021-11956-6.

## Background

The prevention of youth tobacco use is critical to public health. Susceptibility to tobacco use, defined as the lack of a determined decision not to use tobacco, is a robust predictor of youth tobacco initiation [1]. Understanding the susceptibility to tobacco use helps identify adolescents who are at high risk for tobacco use and can aid the design of prevention strategies. Numerous studies have shown that youth who were susceptible to a tobacco product were more likely to start using that tobacco product. For example, Pierce and colleagues (1996) found that youth never smokers, who were susceptible to smoking were five times as likely to start smoking in the subsequent 4 years compared to those who were not susceptible [1]. In the past decade, e-cigarettes have become the most commonly used tobacco products- among youth in the United States (US) [2, 3]. Several recent studies have found that youth who were susceptible to e-cigarette use at baseline were more likely to start using e-cigarettes during follow-up [4, 5]. These studies provide product-specific evidence for the susceptibility-use relationship.

Recently, mounting evidence has suggested that the susceptibility-use relationship may not be product specific. For example, Chaffee and Cheng studied the association between changes in susceptibilities to tobacco use (i.e., curiosity and willingness to try) and the initiation of tobacco use (i.e., never to ever use) among youth never tobacco users and found a wide range of cross-product associations using data from Population Assessment of Tobacco and Health (PATH) wave 1 and 2 surveys [6]. Similar findings have been documented in a longitudinal study of Canadian 9th–12th graders when studying both initiation (never to ever use) and current use [7]. Focusing on e-cigarettes and cigarettes, Nicksic and Barnes (2019) found evidence supporting cross-product prediction [8]. That is, susceptibility to either e-cigarette use or cigarette smoking predicted the initiation of cigarette smoking and the initiation of e-cigarette use (i.e., never to ever use). Further inspection revealed a large degree of overlap in the susceptibility to cigarette smoking and the susceptibility to e-cigarette use (i.e., 67% of youth who were susceptible to cigarette smoking were also susceptible to e-cigarette use) [8]. It is well known that youth tobacco use is characterized by high levels of concurrent use of multiple tobacco products [3, 9]. In a previous study, we found that youth use of various tobacco products may be manifestations of a tendency to use tobacco in general [10]. In this context, it is of interest to query whether the susceptibility-use relationship operates in a product-specific manner or reflects a general tendency towards tobacco use. If the latter, susceptibility to any tobacco product can help identify youth at high risk of tobacco onset.

In the current study, we set out to investigate whether susceptibility to tobacco use is product specific or represents a general tendency towards multiple tobacco product use with the following specific aims: (a) to systematically estimate youth susceptibilities to various tobacco products and the degree of overlap; (b) to estimate associations between selected sociodemographic and behavioral variables and susceptibilities to various tobacco products; and (c) to estimate prospective relationships between susceptibility and the onset of various tobacco products among youth 12–17 years of age living in the US using recent data from a nationally representative longitudinal study. In this study, we used a structural equation modeling approach to simultaneously estimate product-specific relationships and the relationship linking the general level of susceptibility with tobacco onset to gauge the magnitude of each relationship while accounting for the other. Findings from this study provide insights into the identification of high-risk individuals and can help the design of effective prevention strategies against youth tobacco use.

## Methods

### Study population

In this study, the population of interest is US non-institutionalized civilian adolescents 12–17 years of age who had never used a tobacco product. Data were from the longitudinal PATH study wave 4 (Dec. 2016-Jan. 2018) and wave 4.5 (Dec. 2017-Dec. 2018) surveys (public use files) that used a multi-stage sampling method to draw nationally representative samples after Institutional-Review-Board-approved parent consent and youth assent [11]. There were 10,977 never tobacco users (NTU) at wave 4, among whom 8841 were followed up at wave 4.5. In contrast to school surveys of adolescents, the PATH sample includes young people irrespective of school attendance, and its sampling frame includes college dormitories and children of active-duty military living in the US. More details about the PATH methodology are provided elsewhere [11]. PATH Public Use data files were downloaded from https://www.icpsr.umich.edu/icpsrweb/NAHDAP/studies/36498 on Dec. 13, 2019 (wave 4 data) and September 18, 2020 (wave 4.5 data).

### Assessment

Audio computer assisted self-interviews (ACASI) with standardized multi-item modules were used to assess tobacco use history and a range of related variables. Never tobacco users at wave 4 were individuals who had never used any of the tobacco products assessed, even one time. Products assessed included cigarettes, e-cigarettes, cigars (traditional cigars, cigarillos, and filtered cigars), smokeless tobacco, snus, hookah, pipe, dissolvable tobacco, bidis, and kretek. Survey questions about ever use of these tobacco products were typically in the format of “Have you ever smoked/used …, even one or two puffs/times?”

Susceptibility to tobacco use was assessed via the following questions:

- “Have you ever been curious about using/smoking …?”
- “Do you think you will try/smoke … in the next year?”
- “Do you think that you will try/smoke … soon?”
- “If one of your best friends were to offer you a …, would you try/smoke it?”

Sets of these susceptibility questions were asked for cigarettes, cigars, cigarillos, filtered cigars, e-cigarettes, hookah, snus, and smokeless tobacco. Each question was rated on a 4-point Likert scale. In line with the literature, we dichotomized susceptibility into non-susceptible and susceptible [1, 8, 12]. The former group included youth who answered “not at all curious” to the first question and “definitely not” to the other three questions. Any non “definitely not/not at all” answer to any of these four questions qualified the youth as susceptible to use of the tobacco product assessed. These measures were adapted from validated items of susceptibility to cigarette smoking [1, 12, 13].

Incident use of a tobacco product was defined as using the product for the first time (i.e., ever use) between wave 4 and wave 4.5 assessments among never users at wave 4.

Information about sex (male or female), age category (12–14 or 15–17 years of age at baseline), and race/ethnicity is from survey items in the Demographics module. When these items were missing, information from the household screening roster was drawn. Other covariates of interest included cannabis use during the past 30 days, alcohol drinking during the past 30 days, peer tobacco use, school performance during the past 12 months (dichotomized as ‘mostly A’s and B’s’ vs. lower grades), and availability of tobacco at home. Cannabis use included using cannabis (marijuana, hash, THC, grass, pot, or weed) as well as blunts. Peer tobacco use was assessed via questions worded “how many of your best friends use/smoke …?” Separate questions were asked for cigarettes, e-cigarettes, traditional cigars, cigarillos, filtered cigars, snus, and smokeless tobacco. In this study, we dichotomized peer use into “none” and “any” for each tobacco product category assessed. Cannabis use, alcohol drinking, and peer tobacco use were based on the adolescent’s self-report. School performance and availability of tobacco products at home were based on information about the adolescent provided by a parent/guardian. Supplemental Table S1 provides details about the assessment of these covariates.

### Analysis

First, we estimated the proportion of adolescent NTU who were susceptible to each of the tobacco products. We then used a Venn diagram to visualize the degree of overlap in susceptibility to various tobacco products. To ease the visualization, we categorized tobacco products into three groups – combusted tobacco products (i.e., cigarettes, cigars, hookah), e-cigarettes, and oral tobacco products (i.e., smokeless tobacco and snus).

Next, we estimated the susceptibility to tobacco products by sociodemographic characteristics and behavioral variables.

To assess whether wave 4 susceptibility predicted the onset of tobacco product use, we used generalized linear regression with a log link to estimate incidence ratios for each tobacco product category studied here given by log(*p*) = *β*_0_ + *β*_1_*x*_1_, where *p* is the probability of using a tobacco product for the first time (i.e., incidence of tobacco use) between wave 4 and wave 4.5, *x*_*1*_ is the susceptibility to a tobacco product at wave 4, and *β*_1_ is the estimated log incidence ratio of those who were, and were not, susceptible to tobacco use.

**Fig. 1:**
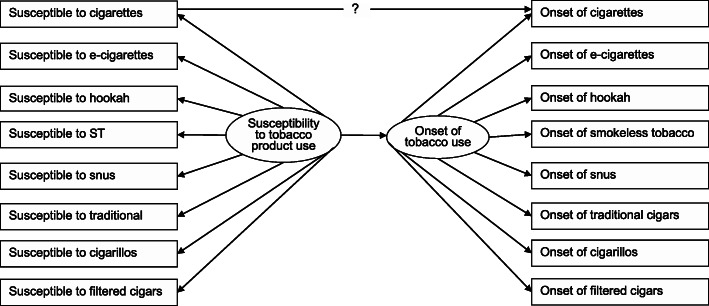
Depiction of a conceptual structural equation model to predict the onset of first cigarette smoking

In the last analysis step, we employed a structural equation modeling (SEM) approach to estimate the product-specific susceptibility-use relationship while accounting for a general susceptibility-use relationship, as depicted in Fig. 1. Structural equation models are multi-equation models that allow simultaneous estimation of relationships of various independent and dependent variables. That is, SEM can incorporate various direct and indirect paths between latent and observed variables based on theories or hypotheses. It is particularly useful when a certain condition (e.g., general openness to tobacco use) cannot be directly observed but can be derived from a set of observable behaviors that often co-occur because of the underlying construct. As shown in Fig. 1, we conceptualized a latent construct for susceptibility to tobacco use (i.e., the general openness to tobacco use) which gave rise to the susceptibility to various tobacco products. We also conceptualized a latent construct for tobacco use onset that represents the level of liability to starting tobacco use in general. In the model depicted in Fig. 1, the estimate of the direct path leading from the susceptibility to cigarette smoking to the onset of cigarette smoking presents the cigarette-specific susceptibility-onset relationship after accounting for the general level of susceptibility to tobacco use and the general level of tobacco onset.

Measurement models were evaluated before the structural paths were drawn. Multiple fit indices were used to evaluate the goodness of fit, including root mean square of approximation (RMSEA) [14], comparative fit index (CFI) [15], and Tucker-Lewis index (TLI). A RMSEA< 0.08 and CFI/TLI > 0.90 were considered as indications of reasonably good model fit [16, 17].

All analyses were weighted. Wave 4 cross-sectional weights were used for wave 4 cross-sectional analysis. Wave 4.5 longitudinal weights for wave 4 cohort were used for wave 4 and 4.5 prospective analysis. These weights incorporate adjustment for selection probability, nonresponse patterns, possible deficiencies in the sampling frame, and attrition [11]. Variances of estimates were produced using balance repeat replication methods (Fay’s method with Fay = 0.3). A robust weighted least square mean and variance (WLSMV) adjusted estimator, which uses a full weight matrix, was used to accommodate categorical variables and the complex survey design in structural equation models. Analyses were conducted using Stata 16.0 (StataCorp, College Station, Texas, USA) and Mplus 8.1 (Muthén & Muthén, Los Angeles, CA, USA).

## Results

### Susceptibility to tobacco products among never users

**Fig. 2:**
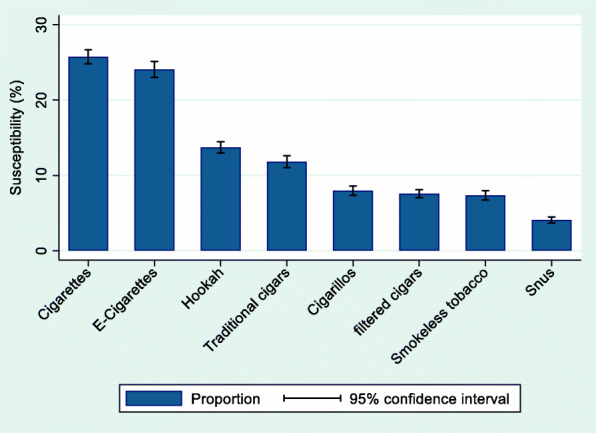
Estimated susceptibility to various tobacco products among youth never tobacco users

At wave 4, there were a total of 10,977 NTU, of whom 50% were girls, 59% were 12–14 years of age, 52% were non-Hispanic White, 14% were non-Hispanic Black, 23% were Hispanics, and 10% were other race/ethnicity groups. Most youth NTU (63%) were not susceptible to any tobacco use. (For all susceptibility measures, there were < 1% missing values.) As shown in Fig. 2, cigarettes and e-cigarettes were the most common tobacco products that youth were susceptible to, and snus was the least common with less than 5% of youth never users susceptible to snus use.

**Fig. 3:**
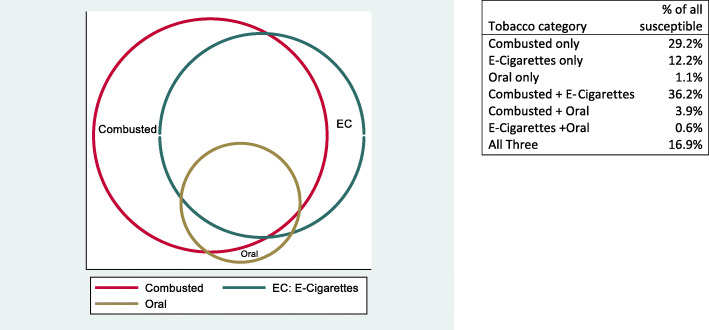
Relations of susceptibility to combusted tobacco products, e-cigarettes, and oral tobacco products

The Venn diagram revealed a large degree of overlap in susceptibilities to the three tobacco product categories (see Fig. 3). Fifty-eight percent of youth NTU who were susceptible to any tobacco use were susceptible to at least two categories. When examined at the individual product level, 65% of youth never users who were susceptible to any tobacco use were susceptible to more than one product.

### Characteristics of youth who are susceptible to each and any tobacco product

With respect to demographic characteristics, older adolescents (15–17 years of age) were more likely to be susceptible to all tobacco products assessed compared to younger adolescents (12–14 years of age, Table 1). With respect to behavioral and environmental variables assessed, adolescents who were current cannabis users and/or alcohol drinkers were more likely to be susceptible to tobacco use (Table 1). In addition, those who affiliated with peers who used tobacco were more likely to be susceptible to tobacco use (Table 1). For other variables, the association was not consistent across tobacco products. For example, males were more likely than females to be susceptible to traditional cigars, hookah, snus, and smokeless tobacco, but not for cigarettes, cigarillo, filtered cigars, and e-cigarettes. Non-Hispanic Black youth were more likely to be susceptible to cigarillo and hookah use compared to non-Hispanic White youth; Hispanic youth were also more likely to be susceptible to hookah use compared to non-Hispanic White youth; non-Hispanic White youth were more likely to be susceptible to smokeless tobacco use compared to non-Hispanic Black and Hispanic youth. Youth with mostly A’s and B’s were less likely to be susceptible to cigarettes, cigarillos, and filtered cigars compared to those with lower academic achievement, whereas no statistically significant differences were observed for other tobacco products under study (Table 1).

**Table 1:** Susceptibility (%) to tobacco product use among youth never tobacco users

Panel A. Susceptibility (%) to tobacco product use by selected demographic characteristics															
		Sex			Age			Race/Ethnicity							
	Total	Male	Female	*p*-value	12–14	15–17	*p-*value	Non-Hispanic White	Non-Hispanic Black		Hispanic	Others	*p-*value		
Cigarette	26	25	26	0.087	**24**	**29**	**< 0.001**	25	24		27	27	0.210		
Cigarillos	8	8	8	0.318	**7**	**9**	**0.001**	**7**	**12**		**8**	**8**	**< 0.001**		
Traditional Cigars	12	**14**	**10**	**< 0.001**	**9**	**15**	**< 0.001**	13	12		10	12	0.075		
Filtered Cigars	8	8	7	0.065	**7**	**9**	**< 0.001**	**7**	**8**		**8**	**8**	0.828		
Hookah	14	**12**	**16**	**< 0.001**	**10**	**19**	**< 0.001**	**12**	**17**		**15**	**14**	**< 0.001**		
E-Cigarette	24	24	24	0.934	**20**	**30**	**< 0.001**	24	23		24	25	0.797		
Snus	4	**5**	**4**	**0.028**	**3**	**5**	**0.001**	**4**	**2**		**4**	**5**	**0.003**		
Smokeless Tobacco	7	**8**	**6**	**< 0.001**	**7**	**8**	**0.029**	**9**	**4**		**5**	**7**	**< 0.001**		
Any Tobacco	37	36	37	0.479	**32**	**44**	**< 0.001**	36	39		37	38	0.207		
Panel B. Susceptibility (%) to tobacco product use by selected psychosocial characteristics															
	Mostly A’s or B’s			Tobacco available at home			Past 30-day marijuana use			Past 30-day alcohol use			Peer tobacco use^a^		
	No	Yes	*p*	No	Yes	*p*	No	Yes	*p*	No	Yes	*p*	No	Yes	*p*
Cigarettes	**28**	**25**	**0.030**	**25**	**33**	**< 0.001**	**26**	**43**	**< 0.001**	**24**	**50**	**< 0.001**	**23**	**46**	**< 0.001**
Cigarillos	**10**	**7**	**< 0.001**	**8**	**10**	**0.043**	**7**	**18**	**< 0.001**	**8**	**20**	**< 0.001**	**7**	**28**	**< 0.001**
Traditional Cigars	12	12	0.512	11	14	0.057	**11**	**30**	**< 0.001**	**12**	**25**	**< 0.001**	**–**	**–**	
Filtered Cigars	**9**	**7**	**0.043**	7	9	0.231	**7**	**17**	**< 0.001**	**7**	**16**	**< 0.001**	**–**	**–**	
Hookah	13	14	0.577	14	15	0.384	**13**	**35**	**< 0.001**	**13**	**35**	**< 0.001**	**–**	**–**	
E-Cigarettes	25	24	0.316	**23**	**32**	**< 0.001**	**24**	**53**	**< 0.001**	**23**	**52**	**< 0.001**	**19**	**53**	**< 0.001**
Snus	5	4	0.059	4	5	0.185	**4**	**11**	**< 0.001**	**4**	**11**	**< 0.001**	**4**	**17**	**< 0.001**
Smokeless Tobacco	8	7	0.388	**7**	**9**	**0.037**	**7**	**13**	**< 0.001**	**7**	**16**	**< 0.001**	**6**	**22**	**< 0.001**
Any Tobacco	**39**	**36**	**0.021**	**36**	**47**	**< 0.001**	**36**	**69**	**< 0.001**	**35**	**69**	**< 0.001**	**30**	**62**	**< 0.001**

Data from PATH wave 4 youth survey 2017–2018 (*n =* 10,977)

Sample size may vary slightly due to missing values

Bold font indicates statistical significance at 0.05 level using a design-adjusted F-test

^a^Peer tobacco use is product specific. For example, peer tobacco use is peer cigarette use for susceptibility to cigarettes. For susceptibility to any tobacco products, peer tobacco use is peer use of any tobacco use assessed (including cigarettes, e-vapor, cigarillos, snus, and smokeless tobacco). Peer use of traditional cigars, filtered cigars, and hookah was not assessed

### Prediction of susceptibility of the onset of tobacco product use

#### Bivariate product-specific prediction

The onset of tobacco use was below 5%, irrespective of whether adolescents were susceptible or not for all tobacco products, except for e-cigarettes (Table 2). Adolescents who were susceptible to a tobacco product were more likely to use the product for the first time within the next year (except for filtered cigars, for which the association was not statistically significant). For example, 18.5% of adolescent NTU who were susceptible to e-cigarettes at wave 4 assessment used e-cigarettes for the first time by the time of the wave 4.5 assessment, whereas 4.3% of those who were *not* susceptible to e-cigarettes did so (incidence ratio = 4.3, 95% CI = 3.6 to 5.0; Table 2). The strongest predictions were seen for smokeless tobacco and cigarillos. Compared to youth who were not susceptible to use smokeless tobacco, those who were susceptible at wave 4 were 12.9 (95% CI = 6.4, 26.1) times likely to use smokeless tobacco for the first time by wave 4.5 follow-up. For cigarettes, youth who were susceptible were 3.2 (95% CI = 2.3, 4.6) times likely to smoke a cigarette by the time of follow-up.

**Table 2:** Onset of tobacco product use (%) and estimated incidence ratio among youth never tobacco users

	Not susceptible	Susceptible	IR^**a**^ (95% CI)	Beta^**b**^ (95% CI)
Cigarettes	1.1	3.6	**3.2 (2.3, 4.6)**	−0.04 (−0.13, 0.05)
Cigars	0.1	0.9	**6.7 (2.5, 18.4)**	0.01 (− 0.15, 0.17)
Cigarillos	0.4	4.2	**9.4 (5.6, 16.0)**	0.06 (− 0.01, 0.13)
Filtered cigars	0.1	0.3	3.8 (0.5, 27.9)	**−0.09 (− 0.13, − 0.05)**
Hookah	0.4	1.6	**3.9 (1.9, 8.1)**	−0.03 (− 0.15, 0.09)
E-Cigarettes	4.3	18.5	**4.3 (3.6, 5.0)**	**0.08 (0.04, 0.13)**
Snus	0.2	1.6	**7.6 (2.4, 24.3)**	−0.02 (−0.17, 0.14)
Smokeless Tobacco	0.2	2.6	**12.9 (6.4, 26.1)**	0.01 (−0.11, 0.12)

^a^IR: incidence ratio estimated using generalized linear regression with a log link

^b^Beta coefficients for the product-specific path were from the structural equation model, in which the estimate linking the susceptibility latent construct and the tobacco onset latent construct was 0.50 (95% CI = 0.42 to 0.58)

Bold font indicates statistical significance at 0.05 level

Data from PATH wave 4 and 4.5 youth surveys (*n =* 8841)

### Results from the structural equation model

Confirmatory factor analysis showed reasonable goodness of fit of a one-factor model that a single construct of susceptibility gives rise to the susceptibility to various tobacco products (RMSEA = 0.052, CFI = 0. 991, and TLI = 0.988), as well as the uni-dimensionality of the onset of tobacco use (RMSEA = 0.019, CFI = 0. 950, and TLI = 0.931) among wave 4 NTU who were followed up at wave 4.5 (*n* = 8841).

In the next steps, a series of structural equation models, as depicted in Fig. 1, were fit to the data to estimate the path for each specific tobacco product. These models fit the data reasonably well (RMSEA< 0.05, CFI > 0. 90, and TLI > 0.90). The susceptibility latent construct was a robust predictor of the latent tobacco onset construct (β = 0.50, 95% CI = 0.42 to 0.58). Estimates of the product-specific path were not statistically significant after accounting for the path between general susceptibility and general tobacco onset (Table 2), except for e-cigarettes (positive prediction: β = 0.08, 95% CI = 0.04 to 0.13) and filtered cigars (inverse prediction: β = − 0.09, 95% CI = − 0.13 to − 0.05). Nonetheless, the magnitude of these product-specific estimates was much smaller compared to the estimate linking the two latent constructs.

## Discussion

Results of these analyses showed that youth susceptibility to tobacco product use typically presents as a general openness to tobacco use rather than product-specific susceptibility. First, we found a substantial overlap in susceptibility across different tobacco products. Second, this general susceptibility to tobacco use coincides with other risk-taking behaviors, such as current use of alcohol and cannabis and socializing with peers who use tobacco. Lastly, the prediction linking susceptibility to tobacco use onset operates primarily at the general tobacco susceptibility level. These findings indicate that a holistic approach towards adverse youth risk behaviors may be more effective in identifying youth who are at high-risk for tobacco use compared to any product-specific approaches.

The substantial overlap in susceptibility to tobacco use observed in this study extended previous evidence on susceptibility to e-cigarette and cigarettes [8] by including various forms of tobacco products and found that the majority (65%) of youth susceptible to using one tobacco product were susceptible to using multiple tobacco products. This finding is in line with a previous study documenting heightened susceptibility to a range of other tobacco products among ever users of a tobacco product compared to nonusers [18]. In addition, youth who used alcohol or cannabis and those who had tobacco-using peers were more likely to be susceptible to tobacco use. These findings suggest that risky behaviors, including the use of other psychoactive substances and peer affiliation, can serve as indicators for youth at high risk for tobacco use. Concordant with previous studies showing cross-category prediction - youth never users who were susceptible to tobacco use were more likely to start using tobacco, alcohol, and other psychoactive drugs compared to youth who were not susceptible to tobacco use [8, 19]. The Common Liability Theory may best explain risky behaviors as it accounts for socio-cultural, structural, and heritable traits, emphasizing individual liability rather than product-specific attributes as contributing to substance use [20]. Some recent studies have highlighted the role of an underlying liability towards tobacco use [10, 21]. The current study extends existing literature on tobacco use behavior to the susceptibility to tobacco use.

Historically, males and individuals with poorer school performance have been found to be more likely to use or be susceptible to tobacco use [1, 8, 22, 23]. In this study, we found that the associations between tobacco susceptibility and sex, race/ethnicity, and school performance were not consistently present for all tobacco products, suggesting that these demographic differences may change with the type of tobacco product. These findings align with published work on tobacco use (Wang et al., 2019) showing that demographic differences varied across tobacco product categories among youth tobacco users [9]. It is important for teachers, parents, and public health professionals to consider these variations in their tobacco prevention efforts as the youth tobacco use landscape can change rapidly. In contrast, use of other psychoactive drugs and peer tobacco use were consistently associated with tobacco susceptibility across all tobacco products studied here. Use of other psychoactive drugs and peer tobacco use may be indicators of risk-taking or novelty-seeking, which also give rise to tobacco susceptibility. Taken together, our findings suggest that indicators of risky behaviors, such as use of other psychoactive drugs or peer tobacco use, may be better predictors for youth tobacco susceptibility compared to demographic characteristics, which may change with social context.

Building upon established findings on product-specific and cross-product prediction [4, 6–8], we found that shared susceptibility is a key predictor of the onset of tobacco use. That is, even though product-specific predictions were strong when studied bivariately, they became relatively small and not statistically robust once the link between general susceptibility and general tobacco onset was considered. In this study we observed a weak but statistically robust product-specific path for e-cigarettes, suggesting that there is an e-cigarette-specific prediction that is not completely accounted for, by the general susceptibility-onset relationship for tobacco products. Future studies are needed to understand factors that might contribute to this e-cigarette-specific path. Nonetheless, this path is much weaker compared to the general susceptibility-onset path. Taken as a whole, a general approach toward tobacco prevention supplemented with e-cigarette-specific prevention may maximize the prevention of onset of tobacco use among youth in the US.

Findings from this study should be interpreted with the following limitations in mind. First, the study is observational in nature and does not provide definitive evidence for causal relationships. Second, we included a range of psychosocial variables in this study, but they are by no means exhaustive. Access to tobacco products, risk perceptions, use of other psychoactive drugs, maladaptive emotional and behavioral problems, family environment, neighborhood characteristics, and tobacco policies are all relevant predictors for tobacco use [4, 24–26]. As shown in our results, although approximately the same portions of youth were susceptible to cigarettes and e-cigarettes, the onset of e-cigarettes was much higher compared to the onset of cigarette smoking, which highlights the role of other variables in youth tobacco onset. Future studies with the consideration of a range of variables would help identify the most important predictors for youth tobacco onset. Third, in this study, we studied the relationship linking susceptibility with the onset of use, the first major milestone of tobacco use. Future studies are needed to investigate whether susceptibility predicts continued use. Lastly, peer tobacco use was not assessed for a few tobacco product categories, which precluded the examination of the relationship between susceptibility and peer use for these tobacco products.

Counterbalancing strengths include: (a) a nationally representative sample; (b) the use of ACASI, which helps reduce reporting bias; (c) a prospective design; (d) use of a structural equation modeling approach, which enables a more nuanced view of the relationship linking susceptibility with tobacco onset; and (e) a focus on youth NTU, which provides evidence relevant for prevention programs.

## Conclusions

In this study, we found a large degree of overlap in youth susceptibilities to various tobacco products; susceptibilities to tobacco use coincide with other risk-taking behaviors, such as current use of alcohol and cannabis and socializing with peers who use tobacco; and the prediction linking susceptibility to tobacco use onset operates primarily at the general tobacco susceptibility level. Taken together, our findings suggest that youth tobacco prevention planning may benefit from a holistic approach towards youth risky behaviors.

## Supplementary Information

**Additional file 1: Supplementary Table 1.** PATH wave 4 youth assessment of covariates used in this study. PATH wave 4 youth assessment of covariates used in this study.

**Additional file 2: Supplementary Table 2.** Fit indices of structural equation models. Fit indices of structural equation models.

## Data Availability

The datasets generated and/or analyzed during the current study are available at https://www.icpsr.umich.edu/icpsrweb/NAHDAP/studies/36498

## Abbreviations

ACASI: Audio computer assisted self-interviews
CFI: Comparative fit index
CI: Confidence interval
NTU: Never tobacco user
PATH: Population Assessment of Tobacco and Health
RMSEA: Root mean square of approximation
SEM: Structural equation modeling
TLI: Tucker-Lewis index
US: United States
WLSMV: Weighted least square mean and variance
