# Supplementary material for: Youth susceptibility to tobacco use: is it general or specific?
Source: BMC Public Health. 2021 Oct 21;21:1913. doi: 10.1186/s12889-021-11956-6 (PMC8532300; doi:10.1186/s12889-021-11956-6)
Supplement: Supplementary file 1 — Additional file 1: Supplementary Table 1. PATH wave 4 youth assessment of covariates used in this study. PATH wave 4 youth assessment of covariates used in this study. [file 12889_2021_11956_MOESM1_ESM.docx]

| Supplementary Table 1. PATH wave 4 youth assessment of covariates used in this study. | | | | | |
| --- | --- | --- | --- | --- | --- |
| Variable | PATH variable | Survey question | Response options | Source of information | Variable used in this study |
| School performance | R04_PT0019 | Please look at this list. How would you describe how (Child’s first name) has performed at school in the past 12 months? Would you say (Child’s first name)’s grades are… | 1 = Mostly A's 2 = A's and B's 3 = Mostly B's 4 = B's and C's 5 = Mostly C's 6 = C's and D's 7 = Mostly D's 8 = D's and F's 9 = Mostly F's 10 = Your child's school is ungraded -97777 = Missing due to data removed per respondent request -8 = Don't know -7 = Refused -1 = Inapplicable -9 = Missing - Not ascertained | Parent/guardian | Dichotomized to “mostly A’s and B’s” vs. other grades  Ungraded was coded missing. |
| Availability of tobacco products at home | R04_PT0029 | Do you think any tobacco products or electronic nicotine products (such as e-cigarettes) might be available to [Child's first name] at your home? | 1 = Yes 2 = No -97777 = Missing due to data removed per respondent request -8 = Don't know -7 = Refused -1 = Inapplicable -9 = Missing - Not ascertained | Parent/guardian | Binary ‘yes’ and ‘no.’ |
| Past 30-day alcohol drinking | R04_YX0673 | Have you used alcohol in the past 30 days? | 1 = Yes 2 = No -97777 = Missing due to data removed per respondent request -8 = Don't know -7 = Refused -1 = Inapplicable -9 = Missing - Not ascertained | Respondent | Binary ‘yes’ and ‘no.’ |
| Past 30-day cannabis use | R04_YX0675 | Have you used marijuana, hash, THC, grass, pot or weed in the past 30 days? | 1 = Yes 2 = No -97777 = Missing due to data removed per respondent request -8 = Don't know -7 = Refused -1 = Inapplicable -9 = Missing - Not ascertained | Respondent | Binary ‘yes’ and ‘no.’  A ‘yes’ response to R04_YX0675 and any response of ‘earlier today,’ ‘Not today but sometime in the past 7 days,’ or ‘Not in the past 7 days but sometime in the past 30 days’ in R04_YJ1112TC, R04_YJ1112CG, R04_YJ1112FC was coded as ‘yes.’ |
|  | R04_YJ1112TC | When was the last time you smoked a traditional cigar as a blunt, even one or two puffs? | 1 = Earlier today 2 = Not today but sometime in the past 7 days 3 = Not in the past 7 days but sometime in the past 30 days 4 = Not in the past 30 days but sometime in the past 6 months 5 = Not in the past 6 months but sometime in the past year -97777 = Missing due to data removed per respondent request -8 = Don't know -7 = Refused -1 = Inapplicable -9 = Missing - Not ascertained | Respondent |  |
|  | R04_YJ1112CG | When was the last time you smoked a cigarillo as a blunt, even one or two puffs? |  | Respondent |  |
|  | R04_YJ1112FC | When was the last time you smoked a filtered cigar as a blunt, even one or two puffs? |  | Respondent |  |
| Peer cigarette use | R04_YX0680 | How many of your best friends smoke cigarettes? | 1 = None 2 = A few 3 = Some 4 = Most 5 = All -97777 = Missing due to data removed per respondent request -8 = Don't know -7 = Refused -1 = Inapplicable -9 = Missing - Not ascertained | Respondent | Binary ‘yes’ and ‘no.’ |
| Peer e-cigarette use | R04_YX0681 | How many of your best friends use e-cigarettes or other electronic nicotine products? |  | Respondent | Binary ‘yes’ and ‘no.’ |
| Peer cigarillo use | R04_YX0683 | How many of your best friends smoke cigarillos? |  | Respondent | Binary ‘yes’ and ‘no.’ |
| Peer snus use | R04_YX0693 | How many of your best friends use snus? |  | Respondent | Binary ‘yes’ and ‘no.’ |
| Peer smokeless tobacco use | R04_YX0685 | How many of your best friends use other types of smokeless tobacco (such as dip, spit, or chew)? |  | Respondent | Binary ‘yes’ and ‘no.’ |
