# Supplementary material for: Youth susceptibility to tobacco use: is it general or specific?
Source: BMC Public Health. 2021 Oct 21;21:1913. doi: 10.1186/s12889-021-11956-6 (PMC8532300; doi:10.1186/s12889-021-11956-6)
Supplement: Supplementary file 2 — Additional file 2: Supplementary Table 2. Fit indices of structural equation models. Fit indices of structural equation models. [file 12889_2021_11956_MOESM2_ESM.docx]

| **Supplementary Table 2. Fit indices of structural equation models.** | | | | |
| --- | --- | --- | --- | --- |
| Model with product-specific path | RMSEA (90% CI) | CFI | TLI | Chi-square (df) |
| Cigarettes | 0.022 (0.021, 0.024) | 0.991 | 0.989 | 560.842 (103) |
| E-Cigarettes | 0.022 (0.020, 0.024) | 0.991 | 0.990 | 538.944 (103) |
| Cigars | 0.023 (0.021, 0.024) | 0.991 | 0.989 | 570.303 (103) |
| Cigarillos | 0.022 (0.020, 0.024) | 0.991 | 0.990 | 554.420 (103) |
| Filtered cigars | 0.021 (0.019, 0.023) | 0.992 | 0.991 | 507.047 (103) |
| Hookah | 0.022 (0.021, 0.024) | 0.991 | 0.989 | 556.800 (103) |
| Snus | 0.023 (0.021, 0.024) | 0.991 | 0.989 | 570.012 (103) |
| Smokeless Tobacco | 0.022 (0.021, 0.024) | 0.991 | 0.989 | 562.978 (103) |
